# Voltage and pH difference across the membrane control the S4 voltage-sensor motion of the Hv1 proton channel

**DOI:** 10.1038/s41598-020-77986-z

**Published:** 2020-12-04

**Authors:** T. Moritz Schladt, Thomas K. Berger

**Affiliations:** 1 Department of Molecular Sensory Systems, Center of Advanced European Studies and Research (Caesar), Bonn, Germany; 2 Department of Neurophysiology, Institute of Physiology and Pathophysiology, Philipps-University Marburg, Marburg, Germany

**Keywords:** Biophysics, Membrane biophysics

## Abstract

The voltage-gated proton channel Hv1 is expressed in a variety of cells, including macrophages, sperm, and lung epithelial cells. Hv1 is gated by both the membrane potential and the difference between the intra- and extracellular pH (ΔpH). The coupling of voltage- and ∆pH-sensing is such that Hv1 opens only when the electrochemical proton gradient is outwardly directed. However, the molecular mechanism of this coupling is not known. Here, we investigate the coupling between voltage- and ΔpH-sensing of *Ciona intestinalis* proton channel (ciHv1) using patch-clamp fluorometry (PCF) and proton uncaging. We show that changes in ΔpH can induce conformational changes of the S4 voltage sensor. Our results are consistent with the idea that S4 can detect both voltage and ΔpH.

## Introduction

The voltage-gated proton channel Hv1 (the product of the HVCN1 gene; also called voltage-sensor domain-only protein, VSOP) is expressed in a variety of organisms, ranging from single-cell organisms to mammals^1–3^. In humans, Hv1 is expressed in several different immune cells^4,5^, lung epithelial cells^6^, and sperm^7^. The physiological role of Hv1 is best understood in macrophages, where the channel regulates the innate immune response to pathogens. Hv1 sustains the production of reactive oxygen species by NADPH oxidase (NOX) activity^4,8,9^, which depolarizes the membrane and acidifies the cytosol. Hv1 activation repolarizes the membrane and counteracts acidification by proton extrusion. In addition, Hv1 has been suggested to regulate B-cell proliferation^5^ , sperm maturation^7^, and the pH at the airway epithelium^6^. Excessive Hv1 activity can have pathological consequences. In a mouse model of ischemic stroke, Hv1 activity worsens brain damage^10^. Hv1 is overexpressed in breast-cancer cells^11^ and malignant B-lymphocytes^12^.

Hv1 is a special member of the family of voltage-gated ion channels. Classical voltage-gated ion channels comprise a voltage-sensor domain (VSD), consisting of four transmembrane segments, S1–S4, and a pore domain (PD), consisting of two transmembrane segments (S5 and S6). Four subunits assemble such that the four PDs form a central pore. In contrast, Hv1 consists only of a VSD, lacking the pore domain^1,2^ . Reconstituted Hv1 is functional in liposomes^13^, showing that the VSD harbors the pore for proton permeation. The intracellular C-terminal end of Hv1 contains a coiled-coil domain that promotes dimer formation^14–17^. Each subunit contains its own ion permeation pathway^14–16^, and the subunits are gated cooperatively^18,19^. Amino-acid residues that affect proton selectivity have been identified^20,21^, providing an initial, albeit incomplete picture of the proton-permeation pathway. The gating process involves an outward motion of the main voltage-sensing segment S4^18^. An additional conformational change has been identified, which involves the S1 segment and which is concomitant with channel opening^22^.

Hv1 is not only gated by membrane potential, but also by the pH difference across the membrane (ΔpH = pH_o_ − pH_i_). At ΔpH = 0, Hv1 starts to open at + 10 to + 30 mV^23^. The activation curve is shifted by approximately 40 mV/∆pH unit to more negative and positive membrane potentials for ΔpH > 0 and < 0, respectively (“40 mV rule”)^24^. By contrast, changes in pH_o_ or pH_i_ that leave ΔpH constant do not alter the voltage dependence of activation. An important functional consequence of ΔpH sensing in Hv1 is outward rectification: the channel only opens when the electrochemical gradient is directed towards the extracellular side (see^25^ for an exception). The amino-acid residues that convey ΔpH sensing remain elusive. Although mutants with enhanced or diminished ΔpH sensing have been identified^26–28^, no mutants are known that lack ΔpH sensing entirely. In summary, coupling between voltage- and ΔpH-sensing in Hv1 is not well understood.

Here, we study the mechanism of ΔpH sensing in Hv1. Using electrophysiological and fluorescence-optical techniques, we show that the S4 voltage sensor of ciHv1 changes its conformation in response to changes in ΔpH. Our results suggest that S4 is not only the main voltage sensor, but that S4 also serves as a ΔpH sensor.

## Methods

### Ethical approval

*Xenopus laevis* frog oocytes were provided by Christopher Volk (Bonn-Rhein-Sieg University of Applied Sciences, Sankt Augustin, Germany), purchased from Ecocyte (Castrop-Rauxel, Germany), or harvested from our own colony. Frogs were housed according to the German law of animal protection and the district veterinary office. Oocytes were harvested from frogs anesthetized in phosphate-buffered water containing 0.16% 3-aminobenzoate methanesulfonate salt. The surgery followed standard procedures and were carried out in accordance with relevant guidelines and regulations with the approval (84–02.04.2016.A077) of the local authority of the state North Rhine-Westphalia (LANUV) or the approval (Nr. A 16/2019) of the local authority of the state Hesse (Regierungspräsidium Gießen).

### DNA constructs and expression in *Xenopus* oocytes

DNA constructs were cloned and sequenced using standard techniques. The psD64TF vector^2^ containing ciHv1 (accession number NP_001071937) was linearized with SacI, and the region coding for ciHv1 was transcribed using the SP6 mMessage mMachine kit (Ambion, Austin, TX, USA). *Xenopus* oocytes were injected with 50 nl RNA (0.1–2 μg/μl) and incubated at 14–16 °C for 1–5 days in ND96 medium containing (in mM): 96 NaCl, 2 KCl, 1.8 CaCl_2_, 1 MgCl_2_, 10 4-(2-hydroxyethyl)piperazine-1-ethanesulfonic acid (HEPES), 5 Na-pyruvate, and 100 mg/l gentamicin, adjusted to pH 7.5 with NaOH.

### Electrophysiological recordings

Prior to recording, oocytes were mechanically devitellinated under a stereoscope and placed in a recording chamber under an inverted IX71 microscope (Olympus, Tokyo, Japan) equipped with a 10 × or 20 × objective. Patch electrodes were pulled from 1.5 mm thick borosilicate glass capillaries (Hilgenberg, Malsfeld, Germany) on a DMZ puller (Zeitz Instruments GmbH, Martinsried, Germany) and subsequently fire polished with a Narishige MF-830 microforge (Narishige, Tokyo, Japan). The resulting initial electrode resistance was 0.6–1.5 MΩ (about 8–30 μm inner tip diameter) in the used recording solutions. Excised macro patches were obtained within seconds to minutes. Holding potentials were − 60 mV or − 80 mV. Recordings were performed at room temperature (RT, 22–25 °C) using an Axopatch 200B amplifier (Molecular Devices, Union City, CA, USA), connected via a Digidata 1440A acquisition board (Molecular Devices) to a PC running the ClampEx software (Molecular Devices). Data were filtered at 2 or 5 kHz and the sampling rate was 10 kHz. Pipette and bath solutions contained (in mM): 100 HEPES, 30 methanesulfonic acid, 5 tetraethylammonium chloride (TEA-Cl), and 5 EGTA, adjusted to pH 7.0 or pH 7.5 with TEA hydroxide (> 25 mM). HEPES was replaced by 2-(N-morpholino)ethanesulfonic acid (MES) in recording solutions adjusted to pH 6.5. Large proton currents generated by the activation of proton channels can lead to accumulation or depletion of protons at either side of the membrane, thereby changing the pH in the vicinity of the proton channel^29^. As a consequence, the electrochemical driving force for protons can change (reduction of outward currents during channel activation), precluding stable recording conditions. In order to keep the pH during Hv1 activation as stable as possible, we used solutions with high pH buffer concentrations (100 mM), used patch pipettes with large tip diameters to obtain a large pH-buffer volume-to-membrane surface ratio, and used oocytes with low Hv1 expression levels to record rather small proton currents. Chemicals were purchased from Sigma-Aldrich (St. Louis, MO, USA), Carl Roth (Karlsruhe, Germany), Thermo Fisher Scientific Inc. (Waltham, MA, USA), Toronto Research Chemical (Toronto, ON, Canada), or Tocris (Bristol, UK).

### PCF recordings

On the day of recording, oocytes were labeled at 4 °C for 45–60 min in a solution containing (in mM): 92 KCl, 0.75 CaCl_2_, 1 MgCl_2_, 10 HEPES, and 0.05 (2-((5(6)-Tetramethyl-rhodamine)carboxylamino)ethyl)methanethiosulfonate (MTS-TAMRA), adjusted to pH 7.5 with KOH. Subsequently, oocytes were washed three times in ND96 and stored at 12 °C until recording. Excised patches were obtained under visual control using a 10× objective. The objective was changed to a 60× oil-immersion objective (Olympus Apo N 60XOTIRF NA1.49 or PLAPON 60XOTIRFM NA 1.45). TAMRA was excited with a Spectra X (Lumencor, Beaverton, OR) at 550/15 nm or a Polychrome 5 light source (Till Photonics, Martinsried, Germany), and the fluorescence emission was monitored through a TRITC filter cube (Semrock, Rochester, NY, USA; excitation FF01-543/22–25, dichroic FF562-Di03, and emission FF01-593/40, or excitation FF01-542/20, dichroic FF570-Di01, and emission FF01-620/52) and detected with an iXon Ultra DU-897U or a Luca S emCCD camera (iXon Ultra, Andor Technologies, Belfast, UK). The frame rate was 200 Hz, 8 × 8 pixels were binned and registered in the frame-transfer mode using the camera’s conventional output amplifier. Acquisition was triggered externally via the ClampEx software. The light intensities, measured with a PS19Q sensor connected to a FieldMax-TOP power meter for visible light (Coherent, Dieburg, Germany) at the level of the recording stage, were ~ 0.16 mW/mm^2^.

### Uncaging of NPE-caged-proton

1-(2-nitrophenyl)ethyl (NPE)-caged-proton (Tocris, Bristol, UK) was dissolved in DMSO and added to the pipette solution at a final concentration of 500 µM (1% DMSO), just prior to the experiment. The pipette solution contained (in mM): 0.1 HEPES, 90 NMDG, 30 methanesulfonic acid (MS), 5 TEA chloride, and 5 EGTA adjusted to pH 7.5 with MS. NPE-caged-proton was photolysed with a UV LED (365 nm, Thorlabs, Newton, New Jersey, United States). The light stimulus was triggered by the pClamp software. The light intensity, measured with a power meter at the level of the recording stage, was ~ 0.86 mW/mm^2^. In PCF experiments combined with uncaging, MTS-TAMRA was excited by a Spectra X light source at 550/15 nm and the emission filtered with two dichroic 562 LP filter.

### Data analysis

Data were analyzed with Igor Pro (Wavemetrics, Portland, OR, USA). Conductance-voltage relationships (GV) were obtained from normalized tail currents (I_tail_) measured 5–25 ms after the end of the depolarizing voltage step and fitted with the Boltzmann equation: G/G_max_ = 1/(1 + exp(− (V − V_1/2_)/S)), where G_max_ is the maximal conductance, S the slope, V the membrane voltage, and V_1/2_ the voltage at which 50% of the maximal amplitude is reached. From the PCF image frames, the mean (i.e. spatial average) fluorescence intensity was calculated from the brightest pixels (around 5–30 binned pixels, marked with stars in Fig. 2F), and the dark count of the camera was subtracted to obtain the fluorescence F. F was then normalized to the initial baseline level at − 80 mV to obtain ∆F/F. For the average fluorescence traces in different pH conditions (Figs. 2G, 3B, 5C and 6B), only traces in steady-state conditions (i.e. when solution exchange was completed) were used. The voltage-induced change in fluorescence is denoted as F_signal_, and the amplitude of F_signal_ is reported as the difference between the fluorescence at − 80 mV and the steady-state fluorescence at the end of the voltage step. Activation time constants of current (I) and fluorescence (F) were obtained from double-exponential fits, and the deactivation time constants of F were obtained from mono-exponential fits. The minimum of F was taken as starting point for the deactivation. The relationship between voltage dependence or kinetics and pH or ΔpH was tested with linear regression analysis and tested for significant deviations from a zero-slope line with a significance level of α = 0.05. For ciHv1-I175C-TAMRA, the ΔpH dependence of fast and slow activation time constants of F was compared to the respective slopes of fast and slow activation time constants of I by two-tailed paired t tests with a significance level of α = 0.05. r^2^ denotes the coefficient of determination. The ∆pH-induced effects on the F_signal_ amplitude and F(− 80 mV) were tested with one-way ANOVA, followed by Tukey’s test *post-hoc* analysis. All values are reported as mean ± SD.

## Results

### Voltage dependence of Hv1 is sensitive to ΔpH, but not to pH itself

**Figure 1:**
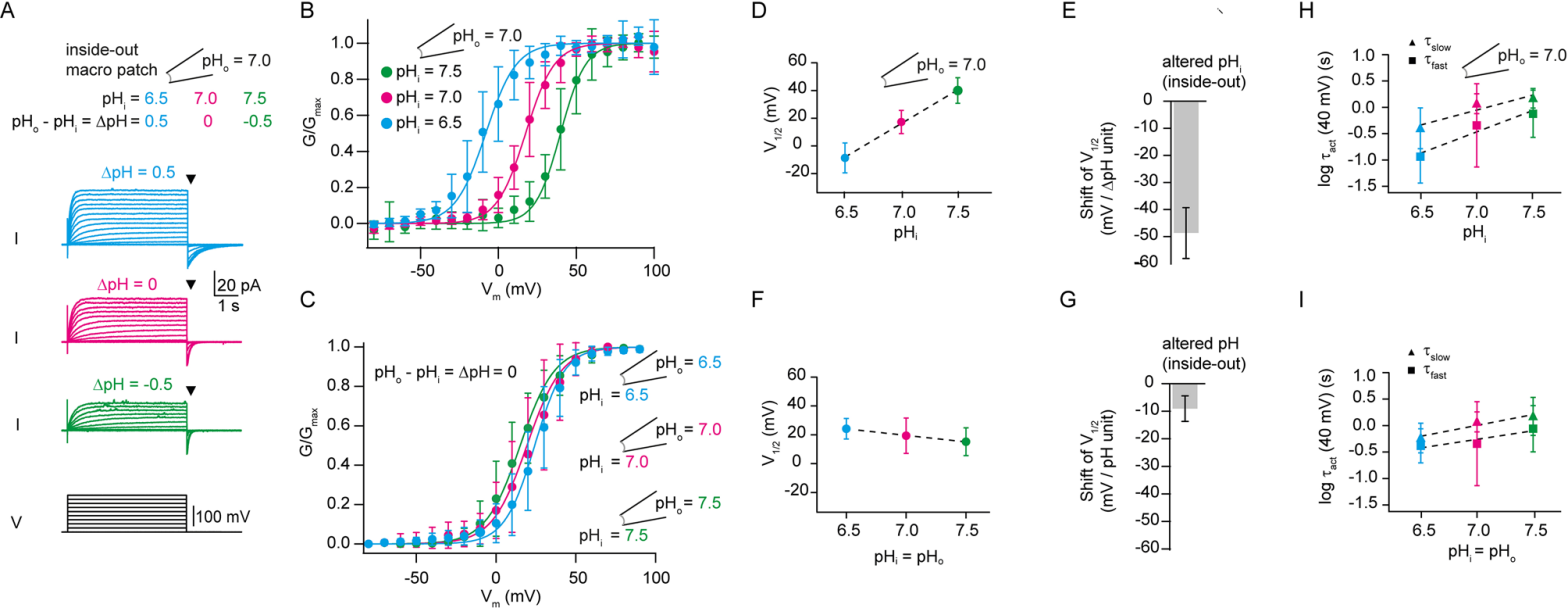
Voltage dependence of ciHv1 is coupled to the difference between pH_i_ and pH_o_ (∆pH), but not to pH itself. (**A**) inside-out patch-clamp recordings of ciHv1 at different ∆pH conditions. (**B**) Mean GVs derived from tail currents (at time points specified by triangles in panel A) at different ∆pH conditions. Data from individual patches were fitted with Boltzmann functions (not shown), and the resulting mean slopes and mean V_1/2_ values were used to construct Boltzmann fits for the mean GVs (see Table 1 for fit parameters). (**C**) GVs derived from tail currents of inside-out patch-clamp recordings of ciHv1 at different pH, leaving ∆pH = 0, fitted with Boltzmann functions. (**D**) V_1/2_ as a function of pH_i_ while pH_o_ = 7. The dashed line is a linear fit with a slope of − 48.6 mV/∆pH unit; r^2^ = 0.8, *p* < 0.05. (**E**) shift of V_1/2_ per ∆pH unit (altered pH_i_, − 48.6 ± 9.5 mV). (**F**) V_1/2_ as a function of the pH itself. The dashed line is a linear fit with a slope of − 9.1 mV/pH unit; r^2^ = 0.1, n.s. **(G)**, shift of V_1/2_ per pH unit (− 9.1 ± 4.6 mV). (**H**) activation time constants τ_fast_ and τ_slow_ as function of pH_i_ while pH_o_ = 7 (see also Table 2). The dashed lines are linear fits with slope(τ_fast_) =  − 0.8 log(s)/ΔpH unit, r^2^ = 0.3, *p* < 0.05, and slope(τ_slow_) =  − 0.6 log(s)/ΔpH unit, r^2^ = 0.4, *p* < 0.05. **(I**), activation time constants τ_fast_ and τ_slow_ as function of pH (∆pH = 0, see also Table 2). The dashed lines are linear fits with slope(τ_fast_) = 0.3 log(s)/pH unit, r^2^ = 0.2, *p* < 0.05, and slope(τ_slow_) = 0.4 log(s)/pH unit, r^2^ = 0.3, *p* < 0.05. Error bars indicate the SD.

To study the coupling between voltage- and ΔpH-sensing, we heterologously expressed Hv1 from *Ciona intestinalis* (ciHv1) in *Xenopus laevis* oocytes. CiHv1 displays robust heterologous expression^18^ and faster activation kinetics than human Hv1, which facilitates data acquisition. Hv1 currents were recorded from excised inside-out membrane patches under various pH conditions (Fig. 1). Voltage steps of increasing amplitudes (up to + 100 mV) gave rise to outward currents (Fig. 1A). From the tail currents (Fig. 1A, arrows), the normalized conductance-voltage relationship (GV) was calculated (Fig. 1B, see methods). We tested the action of ∆pH on the GV relation by changing pH_i_. When pH_i_ was changed from 7.0 to 6.5 (ΔpH = 0.5) or from 7.0 to 7.5 (ΔpH =  − 0.5), the voltage of half-maximal activation (V_1/2_) of the GV relation shifted towards more negative or positive potentials, respectively (Fig. 1B). Similar to previous reports^1,2,24^, the V_1/2_ shifted by − 48.6 ± 9.5 mV/ΔpH unit (Fig. 1D,E, Table 1). By contrast, at symmetrical pH (ΔpH = 0), V_1/2_ shifted only by − 9.1 ± 4.6 mV/pH unit between pH 6.5 and 7.5 (Fig. 1C,F,G), indicating that within this pH range, the voltage dependence of ciHv1, like the voltage dependence of its orthologues from human and mice, is relatively insensitive to pH itself (Fig. 1G). Similar to the ΔpH-dependent changes in kinetics seen in voltage-gated proton currents in rat alveolar epithelial cells^24^, we also find ΔpH-dependent activation kinetics of ciHv1: the fast and slow activation time constants τ_fast_ and τ_slow_ become faster when ΔpH becomes more positive, i.e. when pH_i_ < pH_o_ (Fig. 1H, Table 2). The relationship between log(τ_fast_) and ΔpH, and between log(τ_slow_) and ΔpH was linear in the investigated range. As previously reported for human Hv1^30^, we also find that the activation time constants of ciHv1 are faster at acidic symmetric pH than at alkaline symmetric pH (Fig. 1I and Table 2). However, the dependence of channel activation kinetics on ΔpH is steeper than the dependence of channel activation kinetics on symmetric changes in pH itself (compare Fig. 1H,I). Taken together, the activation kinetics suggest that channel gating depends more on pH_i_ than on pH_o_.

**Table 1:** Fit parameters of the GV relationships of ciHv1 and ciHv1-L245C-TAMRA (mean ± SD).

Channel	V_1/2_ shift/ ∆ pH unit (mV)	pH_i_/pH_o_	V_1/2_ (mV)	Slope (mV)	n
ciHv1	− 48.6 ± 9.5	6.5/7.0	− 8.2 ± 10.7	9.8 ± 2.5	16
		7.0/7.0	17.5 ± 8.0	9.2 ± 3.0	17
		7.5/7.0	39.9 ± 9.0	8.1 ± 3.3	16
		6.5/6.5	24.2 ± 7.1	9.4 ± 2.4	6
		7.5/7.5	15.1 ± 9.6	10.8 ± 1.8	5
ciHv1-L245C-TAMRA	− 54.1 ± 4.8	6.5/7.0	− 50.5 ± 9.5	7.6 ± 1.9	5
		7.0/7.0	− 7.0 ± 12.2	7.4 ± 2.1	8
		7.5/7.0	− 2.5 ± 13.7	6.9 ± 2.2	7

**Table 2:** Activation kinetics (τ_fast_ and τ_slow_) of the current of ciHv1 during a voltage step to 40 mV at different pH_i_ and pH_o_ (mean ± SD).

pH_i_/pH_o_	τ_fast_ (s)	τ_slow_ (s)	n
6.5/7.0	0.16 ± 0.08	0.54 ± 0.44	7
7.0/7.0	0.87 ± 0.69	1.28 ± 0.55	9
7.5/7.0	1.03 ± 0.63	1.58 ± 0.52	6
6.5/6.5	0.49 ± 0.23	0.71 ± 0.36	9
7.5/7.5	1.19 ± 0.83	1.85 ± 1.08	5

### The motion of the S4 segment is similar for different symmetric pH conditions

**Figure 2:**
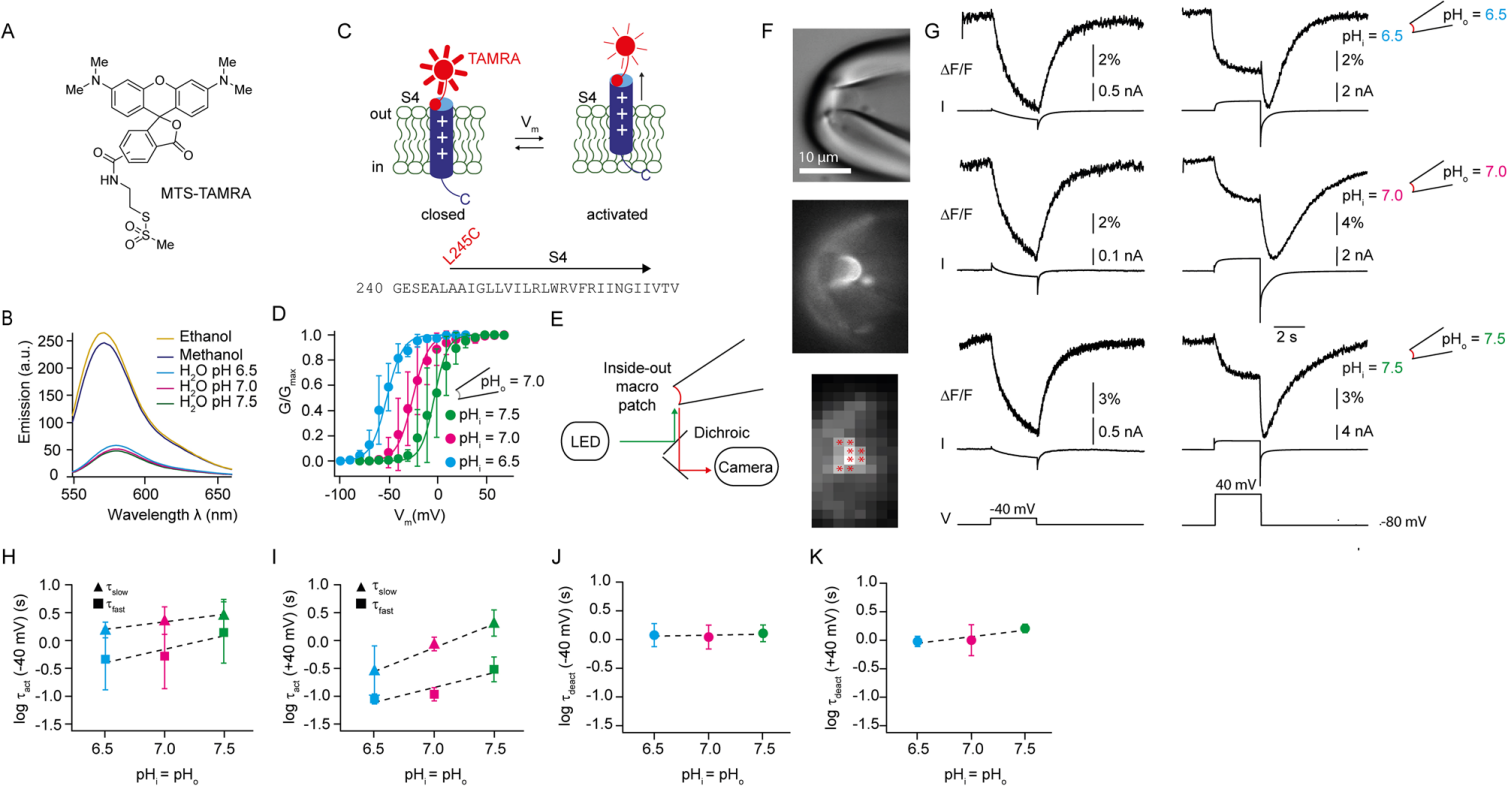
PCF recordings at the extracellular end of S4 for different symmetric pH conditions**.** (**A**) chemical structure of MTS-TAMRA. (**B**) emission spectrum of MTS-TAMRA (50 nM) in ethanol, methanol, and aqueous solutions buffered to various pH values. Excitation wavelength was 542 nm. (**C**) top, cartoon depicting voltage-evoked S4 conformational change of ciHv1-L245C-TAMRA. For clarity, only S4 is shown. “ + ” signs denote the charged arginines in S4. Bottom, amino-acid sequence of the S4 voltage sensor of ciHv1 and the site of labeling. (**D**) GVs derived from tail currents of inside-out patch-clamp recordings of ciHv1-L245C-TAMRA at different ∆pH conditions, fitted with Boltzmann functions (see Table 1 for fit parameters). (**E**) scheme of the inside-out PCF recording condition. (**F**) excised inside-out patch containing ciHv1-L245C-TAMRA (top, bright-field image; middle, epifluorescent image; bottom, 8 × 8-binned epifluorescent image). Red stars mark pixels included in analysis. (**G**) representative inside-out PCF recordings of ciHv1-245C-TAMRA, in response to voltage steps from − 80 to − 40 (left) or + 40 mV (right) at different pH conditions leaving ∆pH = 0. The fluorescence (∆F/F) is the spatial average of the pixel intensities of the marked pixels as exemplified in panel F (bottom, see Methods). (**H**–**I**), mean activation time constants τ_fast_ and τ_slow_ of F_signal_ at − 40 mV (panel H) or + 40 mV (panel I) as a function of pH (see also Table 3). The dashed lines are linear fits with the following slopes: slope(τ_fast_) = 0.5 log(s)/pH unit, r^2^ = 0.2, n.s., and slope(τ_slow_) = 0.3 log(s)/pH unit, r^2^ = 0.1, n.s., at − 40 mV; slope(τ_fast_) = 0.5 log(s)/pH unit, r^2^ = 0.6, *p* < 0.05 and slope(τ_slow_) = 0.9 log(s)/pH unit, r^2^ = 0.5, *p* < 0.05, at + 40 mV. (**J**–**K**) mean deactivation time constants τ_deact_ of F_Signal_ during repolarization from − 40 mV (panel J) or + 40 mV (panel K) to − 80 mV as function of pH (see also Table 3). The dashed lines are linear fits with the following slopes: slope(τ_deact_) = 0.03 log(s)/pH unit, r^2^ = 0.005, n.s., for − 40 mV; slope(τ_deact_) = 0.2 log(s)/pH unit, r^2^ = 0.3, n.s., for + 40 mV. Error bars indicate the SD.

To identify the molecular mechanism of coupling between voltage- and ΔpH-sensing, we studied the underlying conformational changes of Hv1. Previous studies showed that S4 is the main voltage sensor that moves outwardly upon depolarization^18,19,22^. Here, using the PCF technique^22,31,32^, we investigated whether altering ΔpH alone can change the S4 conformation. As readout for conformational changes, we used the thiol-reactive fluorophore MTS-TAMRA (Fig. 2A). TAMRA is environmentally sensitive: the fluorescence intensity in a polar solvent, like H_2_O, is lower compared to that in a less polar solvent, like ethanol or methanol (Fig. 2B). This property has been used to track conformational changes of ion channels^19,33,34^. By contrast, TAMRA is insensitive to changes in pH: aqueous solutions of pH 6.5 to 7.5 do not change the fluorescence intensity (Fig. 2B). Thus, TAMRA is suitable to track protein conformational changes at different pH.

For MTS-TAMRA labeling, a single cysteine was introduced at position L245C, which is located at the extracellular end of S4^35^ (Fig. 2C); this site corresponds to F195C used for labeling of human Hv1^19^. The dependence of V_1/2_ on ∆pH in this labelled mutant, called ciHv1-L245C-TAMRA, is preserved (Fig. 2D and Table 1). Fluorescence in voltage-clamped, excised inside-out macro-patches was recorded (excitation light: 550 nm) with a camera (Fig. 2E). The membrane patch was visible as a curved fluorescent stripe (Fig. 2F). Pixels were binned to allow for fast frame rates (200 Hz), and voltage-evoked changes in the fluorescence of the membrane patch (ΔF) were analyzed. A previous study using voltage-clamp fluorometry identified complex voltage-evoked fluorescence signals that were interpreted as two consecutive S4 conformational changes that lead to Hv1 channel opening^36^. Here, we probed S4 conformational changes by stepping from − 80 to − 40 or + 40 mV such that the Hv1 channels preferentially populate the activated closed state or the activated open state, respectively. Upon a voltage step from a holding potential of − 80 to − 40 mV (activated closed state), the fluorescence of ciHv1-L245C-TAMRA decreased. The change was reversible: upon stepping back to − 80 mV, the fluorescence intensity returned to its original value (Fig. 2G, left). This voltage-evoked fluorescence signal (F_signal_) is consistent with a previous study^36^ and was interpreted as the initial outward S4 motion during voltage sensing. F_signal_ is similar in overall shape for various symmetric pH conditions (pH_i_ = pH_o_ = 6.5, 7.0, or 7.5, thus ΔpH = 0; Fig. 2G, left). The activation kinetics tend to be faster in acidic conditions; the differences were, however, not significant (Fig. 2H). Likewise, the deactivation kinetics do not depend on the pH itself (Fig. 2J).

**Figure 3:**
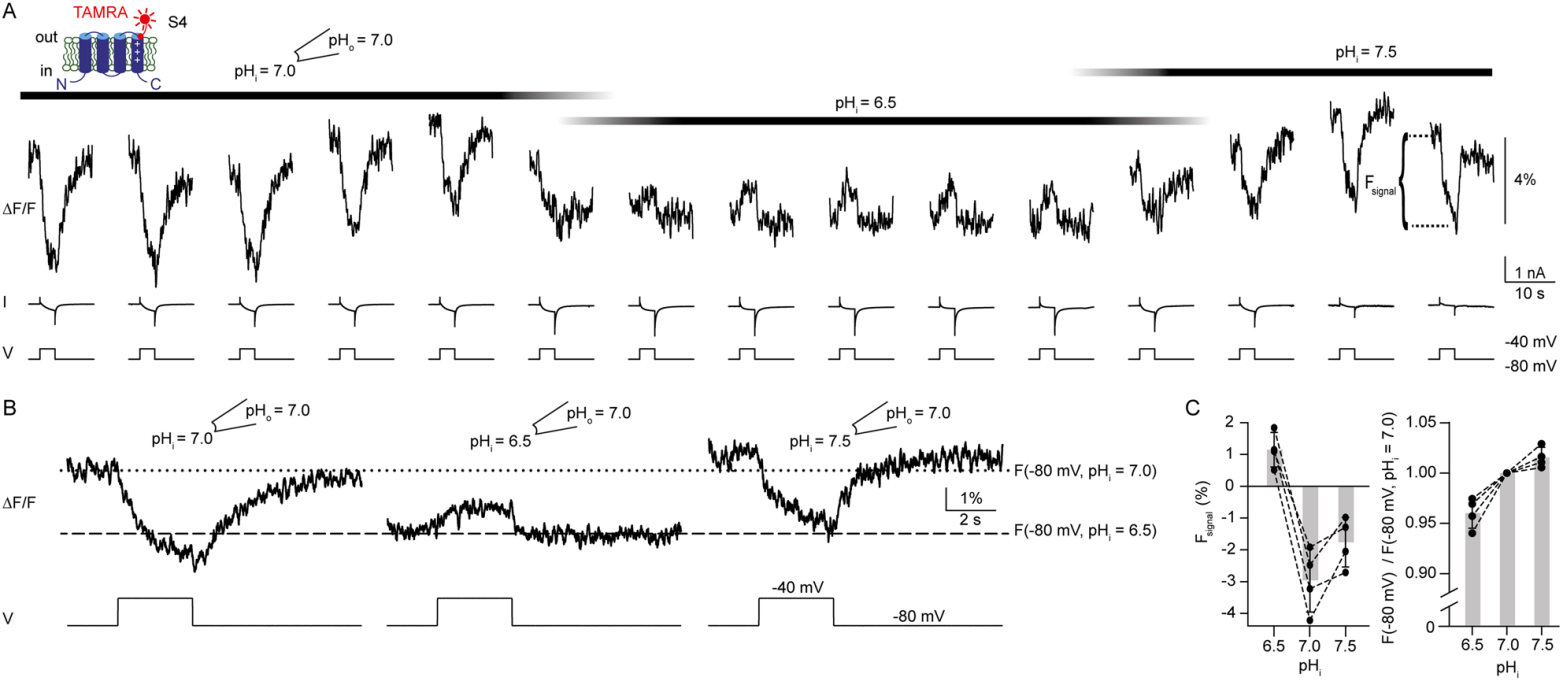
Changes in ∆pH induce S4 conformational changes. (**A**) representative inside-out PCF recording of ciHv1-L245C-TAMRA in response to repetitive voltage steps from − 80 mV to − 40 mV and back while changing pH_i_ and keeping pH_o_ = 7.0. The voltage-evoked fluorescence signal is denoted as F_signal_. (**B**) mean fluorescence signals calculated from (A) for different pH_i_ while pH_o_ = 7.0. Horizontal lines (dotted, pH_i_ = 7.0; dashed, pH_i_ = 6.5) indicate the average fluorescence at − 80 mV. (**C**) left, amplitude of F_signal_ as a function of pH_i_ while pH_o_ = 7.0 (n = 4 patches from 4 different cells). For pH_i_ = 6.5, F_signal_ = 1.1 ± 0.5; for pH_i_ = 7.0, F_signal_ =  − 3.0 ± 1.0; for pH_i_ = 7.5, F_signal_ =  − 1.8 ± 0.8; one-way ANOVA, *p* < 0.001; post-hoc analysis: F_signal_ for pH_i_ = 7.0 vs. F_signal_ for pH_i_ = 6.5: *p* = 0.0001; F_signal_ for pH_i_ = 7.0 vs. F_signal_ for pH_i_ = 7.5: *p* = 0.1; F_signal_ for pH_i_ = 6.5 vs. F_signal_ for pH_i_ = 7.5: *p* = 0.002). Right, baseline fluorescence at − 80 mV (F(− 80 mV)) as a function of pH_i_ while pH_o_ = 7.0, normalized to F(− 80 mV) at pH_i_ = 7.0 (n = 4 patches from 4 different cells). For pH_i_ = 6.5, F(− 80 mV) = 0.96 ± 0.02; for pH_i_ = 7.5, F(− 80 mV) = 1.02 ± 0.01; one-way ANOVA: *p* < 0.001; post-hoc analysis: F(− 80 mV) for pH_i_ = 7.0 vs. F(− 80 mV) for pH_i_ = 6.5, *p* = 0.001; F(− 80 mV) for pH_i_ = 7.0 vs. F(− 80 mV) for pH_i_ = 7.5, *p* = 0.15; F(− 80 mV) for pH_i_ = 6.5 vs. F(− 80 mV) for pH_i_ = 7.5, *p* < 0.001. Error bars indicate the SD.

A voltage step from a holding potential of − 80 to + 40 mV (activated open state) led to a more complex F_signal_: a biphasic decrease of the fluorescence intensity. In addition, upon stepping back to − 80 mV, the fluorescence further decreased and then returned back to baseline, producing a characteristic “hook” in the fluorescence (Fig. 2G, right). Such an F_signal_ was also observed in a previous study^36^ and was interpreted as the result of voltage-dependent Hv1 channel activation from a resting closed state (high fluorescence intensity) via an activated closed state (low fluorescence intensity) to an activated open state (intermediate fluorescence intensity). Like F_signal_ at − 40 mV, F_signal_ at + 40 mV is similar in overall shape at various symmetric pH conditions (pH_i_ = pH_o_ = 6.5, 7.0, or 7.5; thus ΔpH = 0; Fig. 2G, right). However, some kinetics change for different symmetric pH conditions: at + 40 mV, but not at − 40 mV, the fast and slow activation kinetics of F_signal_ are significantly faster at acidic than at alkaline pH (Fig. 2H,I, Table 3). This might suggest that the transition of the intermediate to the open state is dependent on the pH itself. Interestingly, it has been predicted for human Hv1 that intermediate states are particularly dependent on pH_i_^37^. The deactivation kinetics did not change for different symmetric pH conditions (Fig. 2J,K, Table 3). By stepping from − 80 mV to − 40 mV, small inward currents were elicited, which indicates channel opening. However, F_signal_ lacked the characteristic “hook” (Fig. 2G, left and Fig. 3A, B), suggesting that F_signal_ evoked by a voltage step to − 40 mV mainly reports on closed-state channel transitions. This is supported by the opening probability of around 0.2 for ΔpH = 0 at − mV (Fig. 2D), meaning that 20% of the channels were opened and 80% of the channels remained in a non-conducting state.

**Table 3:** Activation (τ_fast_, τ_slow_) and deactivation (τ_deact_) kinetics of the F_signal_ of ciHv1-L245C-TAMRA during a voltage step to − 40 or + 40 mV at different symmetric pH (∆pH = 0; mean ± SD).

pH_i_/pH_o_	Voltage	τ_fast_ (s)	τ_slow_ (s)	τ_deact_ (s)	n
6.5/6.5	− 40 mV	0.77 ± 0.69	1.61 ± 0.56	1.28 ± 0.48	5
	+ 40 mV	0.09 ± 0.02	0.38 ± 0.26	0.97 ± 0.21	3
7.0/7.0	− 40 mV	1.04 ± 1.22	2.67 ± 1.83	1.22 ± 0.59	9
	+ 40 mV	0.11 ± 0.03	0.89 ± 0.25	1.16 ± 0.78	4
7.5/7.5	− 40 mV	2.48 ± 2.37	3.45 ± 2.32	1.35 ± 0.47	9
	+ 40 mV	0.33 ± 0.13	2.34 ± 1.53	1.62 ± 0.28	4

### Motion of the S4 segment is sensitive to changes in ∆pH

We next tested whether changes in ∆pH affect the voltage-dependent motion of the S4 voltage sensor. While recording from an excised inside-out patch containing ciHv1-L245C-TAMRA, we changed ∆pH by switching to solutions of different pH_i_, whereas pH_o_ was kept constant (7.0), and repetitively stepped from – 80 mV to − 40 mV and back (Fig. 3A). For clarity, averages of the amplitudes of F_signal_ from Fig. 3A are summarized in Fig. 3B. When a more acidic solution was washed in (pH_i_ = 6.5, ΔpH = 0.5), the fluorescence under symmetric pH conditions (pH_i_ = pH_o_ = 7, ΔpH = 0) was drastically altered in several aspects: first, the baseline fluorescence intensity was lowered at − 80 mV, indicating that changes in ΔpH affect the S4 conformation in a non-conducting closed state (Fig. 3A, B). Second, the voltage step to − 40 mV induced an increase rather than a decrease of the amplitude of F_signal_ compared to the ∆pH = 0 condition; upon stepping back to − 80 mV, the fluorescence returned to baseline fluorescence intensity (Fig. 3A, B). At more alkaline pH_i_ (pH_i_ = 7.5, ΔpH =  − .5), the baseline fluorescence and F_signal_ resembled those under ∆pH = 0 conditions (Fig. 3A, B). In Fig. 3C, the mean amplitudes of F_ignal_ (left) and the normalized baseline fluorescence (F (− 80 mV), right) at different ∆pH are summarized. Taken together, this data indicates that changes in pH_i_ that introduce a ΔpH > 0 can alter the S4 conformation in a non-conducting state, suggesting that the S4 conformation is not only sensitive to the membrane potential, but also sensitive to changes in ΔpH.

### Proton uncaging in the patch pipette transiently alters pH

**Figure 4:**
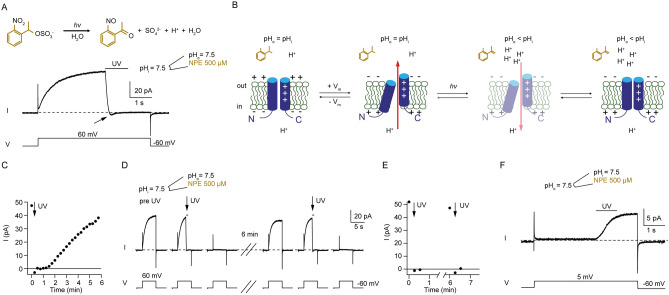
Proton uncaging rapidly and transiently acidifies the pipette solution at the membrane patch. (**A**) top, chemical structure and uncaging reaction of NPE-caged-proton. Bottom, representative inside-out patch-clamp recording of ciHv1 in response to a voltage step from − 60 mV to + 60 mV. UV light was applied for 1 s. The patch pipette contained 500 µM NPE-caged-proton buffered to pH_o_ = 7.5 with 0.1 mM HEPES, while the bath solution was buffered to pH_i_ = 7.5 with 100 mM HEPES. (**B**) gating scheme of ciHv1. For clarity, only S1 and S4 are shown. “ + ” signs denote the charged arginines in S4. Red arrows indicate the direction of proton current. (**C**) current amplitude of an inside-out patch-clamp recording in response to repetitive voltage steps as in A over time (time point indicated by arrow in A). Arrow indicates light stimulus (iteration shown in A). (**D**) representative inside-out patch-clamp recording of ciHv1 in response to voltage steps from − 60 mV to + 60 mV. UV-light stimulation was applied twice to the same patch as indicated by the arrows; the pause between stimulations was approximately 6 min. (**E**) maximal outward current amplitude of the recording in D over time. Arrows indicate iterations with light stimulus. (**F**) representative outside-out patch-clamp recording of ciHv1 in response to a voltage step from − 50 mV to + 5 mV and − 60 mV. UV light was applied for 1 s. The patch pipette contained 500 µM NPE-caged-proton at pH_i_ = 7.5, buffered with 0.1 mM HEPES; pH_o_ was 7.5, buffered with 100 mM HEPES.

So far, we exchanged the pH in the recording chamber by exchanging solution using a gravity-driven perfusion system connected to the recording chamber (Figs. 1B, 2D, 3A). While the exchange of solutions in patch pipettes during a recording is also possible^38^, it is relatively slow and likely to be incomplete. To change the pH (i.e. lower the pH) in the patch pipette during a recording rapidly and without solution exchange (which can introduce artifacts), we used the photolytically cleavable cage 1-(2-nitrophenyl)ethyl sulfate (NPE-caged-proton). NPE-caged-proton releases a sulfate and a proton upon UV irradiation^39^ (Fig. 4A). We tested UV-light induced acidification by uncaging NPE-caged-proton at the extracellular side in excised inside-out patches containing ciHv1. Prior to UV light, robust outward proton currents were recorded in response to voltage steps from − 60 to + 60 mV (Fig. 4A). During depolarization, a 1-s long UV-light stimulus first rapidly diminished the outward current, then led to a brief and small inward current, and finally abolished the current (Fig. 4A). The recording is consistent with a UV-light induced extracellular acidification (and hence a change in the electrochemical driving force for protons) and the coupled ΔpH- and voltage-sensing of Hv1, as depicted in Fig. 4B: depolarization to + 60 mV opens the proton channels and leads to a proton outward current (Transition 1). UV-light induced extracellular acidification changes the electrochemical driving force for protons to such an extent (∆pH <  − 1) that the proton current reverses direction (Transition 2, indicated by arrow in Fig. 4A). The negative ΔpH also closes the proton channels (Transition 3), implying that the open state of ciHv1 is also sensitive to ΔpH. Thus, it is indeed possible to change the pH in the patch pipette during recording. Subsequent depolarizing voltage steps immediately after UV-light application did not elicit proton currents (Fig. 4C). After several minutes, however, the proton-current amplitude recovered (Fig. 4C), showing that the initial pH at the extracellular side of the membrane (facing the lumen of the patch pipette) is reestablished. This suggests that proton uncaging took predominantly place locally in the direct vicinity of the patch membrane (in the focal plane where UV-light intensity is maximal) and not in the bulk volume of the patch pipette, and that subsequent diffusion of buffer and protons reestablishes the initial pH. If this is the case, NPE-caged-protons should diffuse from the bulk volume back to the vicinity of the patch membrane and allow further local uncaging. Indeed, after several minutes, a second UV-light application could again diminish the proton current (Fig. 4D, E). This also shows that the UV-light stimulus does not degrade the functionality of the ciHv1 proton channel. Next, we tested UV-light induced acidification by uncaging NPE-caged-proton at the intracellular side in excised outside-out patches containing ciHv1. Prior to UV light, no or minimal outward proton currents were recorded in response to voltage steps from − 50 to + 5 mV (Fig. 4F). During depolarization, a 1-s-long UV-light stimulus increased the proton-current amplitude significantly (I_pre_ = 0.96 ± 0.9 pA, vs. I_post_ = 5.5 ± 4.1 pA; student´s paired t-test, *p* = 0.01; n = 6) (Fig. 4F), suggesting that uncaging of NPE-caged-proton acidified the intracellular side of the membrane and increased the chemical driving force for protons and increased the opening probability of Hv1. Taken together, UV-light induced uncaging of NPE-caged-proton can rapidly and transiently acidify the pipette solution at the excised membrane patch.

### Motion of the S4 segment can be induced by proton uncaging

**Figure 5:**
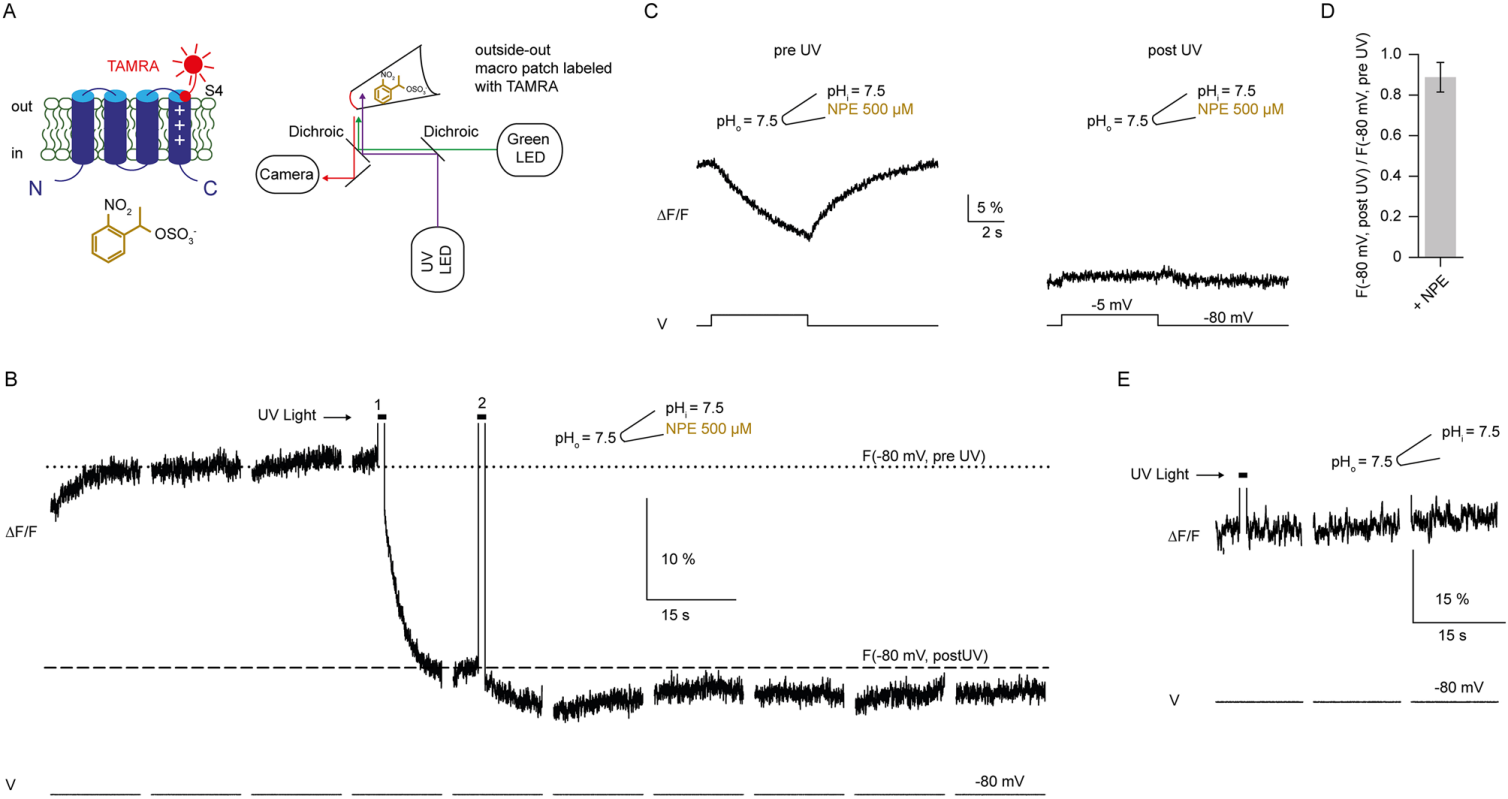
Proton uncaging changes the S4 conformation in closed state. **(A**) left, cartoon depicting ciHv1-245C-TAMRA and chemical structure of NPE-caged-proton at the intracellular side. Right, scheme of the outside-out PCF recording condition with NPE-caged-proton in the pipette. (**B**) representative outside-out PCF recording of ciHv1-L245C-TAMRA held at − 80 mV, and 1 s UV-light exposure at time points indicated by bars. Horizontal lines (dotted = pre UV, dashed = after UV) indicate the fluorescence at − 80 mV. (**C**) mean F_signals_ in response to repetitive voltage steps from − 80 mV to − 5 mV before UV (left) and after UV (right). (**D**) fluorescence ratio, after and before 1 s UV-light exposure, at − 80 mV (0.9 ± 0.07; n = 3 different patches from 3 different cells). (**E**) representative outside-out PCF recording of ciHv1-L245C-TAMRA without NPE in the pipette, held at − 80 mV, and 1 s UV-light exposure at time point indicated by bar. Error bars indicate the SD.

Next, we tested whether changing pH by proton uncaging can also induce conformational changes of the S4 voltage sensor. NPE-caged-proton was uncaged at the intracellular side of an outside-out patch containing ciHv1-L245C-TAMRA while at the same time changes of the fluorescence were recorded with PCF (Fig. 5A). The membrane patch was clamped at − 80 mV throughout the experiment, keeping ciHv1-L245C-TAMRA in a non-conducting, closed state (Fig. 5B). During the 1-s long UV-light stimulus, which bled through to the camera, the fluorescence recording could not be interpreted. After the UV-light stimulus, the fluorescence decreased monotonically to a lower steady-state fluorescence intensity (Fig. 5B,D). In contrast, a UV-light stimulus did not change the fluorescence when the patch pipette did not contain caged protons (Fig. 5E), suggesting that it is not the UV-light stimulus directly but indeed the proton uncaging that is responsible for the change in fluorescence. The mean steady-state fluorescence ratio between before and after the light stimulus at − 80 mV (F(− 80 mV, post UV) / F(–80 mV, pre UV)) decreased (Fig. 5D), which is in agreement with the change in the fluorescence ratio in response to intracellular acidification (ΔpH > 0) by gravity-driven perfusion (Fig. 3C, right). Therefore, acidification by proton uncaging corroborates our results from acidification by gravity-driven perfusion, suggesting that the decrease in fluorescence reports on movement of S4 in response to ΔpH. To test whether F_signal_ is also changed by proton uncaging, proton currents were elicited by depolarizing voltage steps during PCF recordings. The mean F_signal_ (same patch as in Fig. 5B) is shown in Fig. 5C. When pH_i_ was lowered by proton uncaging, F_signal_ was altered similarly as seen with gravity-driven perfusion of a solution with a lower pH_i_: The voltage step to –5 mV induced an increase rather than a decrease of the fluorescence as compared to the ΔpH = 0 condition; upon stepping back to − 80 mV, the fluorescence returned to baseline fluorescence intensity (compare Fig. 5 C with Fig. 3B).Taken together, both methods proton uncaging and gravity-driven perfusion, show that the S4 segment changes its conformation when a ΔpH is applied (by lowering pH_i_).

### S1 gating motion is not uncoupled from channel opening by changes in pH_i_

**Figure 6:**
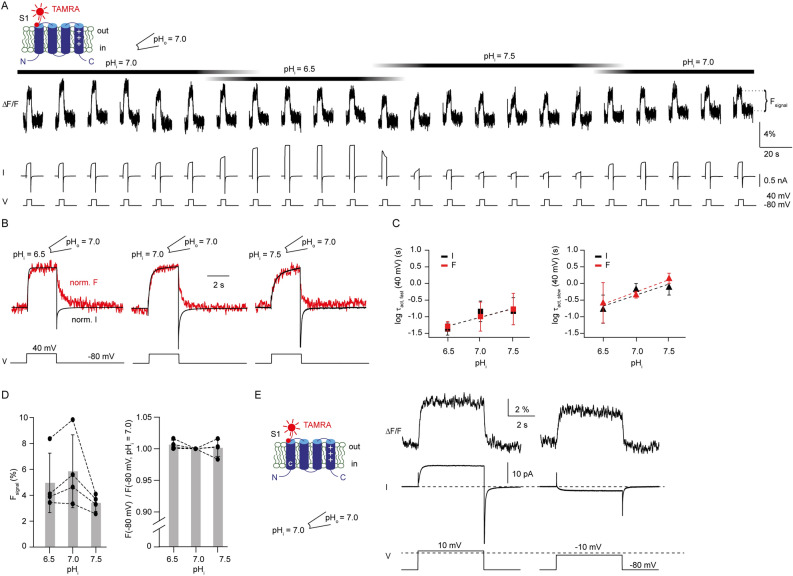
Changes in pH_i_ do not uncouple gating from S1 motion. (**A**) representative inside-out PCF recording of ciHv1-I175C-TAMRA in response to repetitive voltage steps from − 80 mV to + 40 mV and back while changing pH_i_ and keeping pH_o_ = 7.0. (**B**) overlay of normalized mean current and fluorescence derived from the recording in (A). (**C**) mean fast (left) and slow (right) activation time constants of current (black) and F_signal_ (red) of ciHv1-I175C-TAMRA as function of pH_i_ while pH_o_ = 7 (see also Table 4). The dashed lines are linear fits with the following slopes: slope(τ_I fast_) =  − 0.5 log(s)/ΔpH unit, r^2^ = 0.3, n.s.; slope(τ_F fast_) =  − 0.5 log(s)/ΔpH unit, r^2^ = 0.4, *p* < 0.05; slope(τ_I slow_) =  − 0.67 log(s)/ΔpH unit, r^2^ = 0.5, *p* < 0.05; slope(τ_F slow_) =  − 0.73 log(s)/ΔpH unit, r^2^ = 0.5, *p* < 0.05; slope(τ_I fast_) vs. slope(τ_F fast_), n.s.; slope(τ_I slow_) vs. slope(τ_F slow_), n.s. (**D**) left, amplitude of F_signal_ as a function of pH_i_ while pH_o_ = 7.0 (n = 4 patches from 4 different cells). For pH_i_ = 6.5, F_signal_ = 5.0 ± 2.3; for pH_i_ = 7.0, F_signal_ = 5.8 ± 2.8; for pH_i_ = 7.5, F_signal_ = 3.4 ± 0.6; one-way ANOVA, *p* = 0.3. Right, baseline fluorescence at − 80 mV (F(− 80 mV)) for different pH_i_ while pH_o_ = 7.0, normalized to F(− 80 mV) for pH_i_ = 7.0 (n = 4 patches from 4 different cells). For pH_i_ = 6.5, F(− 80 mV) = 1.006 ± 0.007; for pH_i_ = 7.5, F(− 80 mV) = 1.001 ± 0.013; one-way ANOVA, *p* = 0.5. (**E**) representative inside-out PCF recording of ciHv1-I153C-I175C-TAMRA in response to voltage steps from − 80 to + 10 (left) or − 10 mV (right). Dashed line at the bottom indicates 0 mV. Error bars indicate SD.

During pore opening, Hv1 undergoes another conformational change that can be monitored fluorometrically at the extracellular end of S1 (position I175C in ciHv1)^22^. To test whether a change in ΔpH also directly affects S1 motion, we used mutant ciHv1-I175C to label S1 with TAMRA and performed PCF recordings (Fig. 6A). During recording from an excised inside-out patch containing ciHv1-I175C-TAMRA, we changed ΔpH by switching to solutions of different pH_i_, while pH_o_ was kept constant (7.0) and repetitively stepped from − 80 mV to + 40 mV and back (Fig. 6A). To better compare outward currents and fluorescence signals, normalized averages of the data from Fig. 6A are shown in Fig. 6B. As reported previously^22^, in response to a voltage step from − 80 mV to + 40 mV, the S1 F_signal_ and the outward current both increase with similar activation kinetics (Fig. 6A–C, Table 4). In contrast to the S4 F_signal_, the S1 F_signal_ does not change drastically when ∆pH is changed: the baseline fluorescence at − 80 mV did not change and the sign of the S1 F_signal_ remained positive (Fig. 6D). To show that the direction of current does not determine the sign and kinetics of the fluorescence change, we recorded from the double mutant ciHv1-I153C-I175C-TAMRA (Fig. 6E): as shown previously^22^, the I153C mutation shifts the GV relationship to more negative potentials as compared to wild type, so that inward currents can be recorded at negative membrane potentials. The same is true for ciHv1-I153C-I175C-TAMRA, allowing us to record inward currents at − 10 mV and outward currents at + 10 mV. For both conditions, the S1 F_signal_ recorded at position I175C is positive and does not change the sign, confirming that the direction of current does not change the sign of S1 F_signal_. The kinetics of the S1 F_signal_ and the outward current co-varied for different ΔpH: intracellular acidification accelerated and alkalization decelerated kinetics of both the S1 F_signal_ and the outward current (Fig. 6C). Thus, current and S1 F_signal_ kinetics stay coupled even when ΔpH is changed, corroborating that ∆pH- and voltage-sensing are linked to each other via the S4 voltage sensor.

**Table 4:** Activation kinetics of the current and F_signal_ of ciHv1-I175C-TAMRA recorded at pH_o_ = 7.0 (mean ± SD).

pH_i_/pH_o_	τ_I fast_ (s)	τ_F fast_ (s)	τ_I slow_ (s)	τ_F slow_ (s)	n
6.5/7.0	0.05 ± 0.02	0.05 ± 0.01	0.23 ± 0.14	0.44 ± 0.21	4
7.0/7.0	0.17 ± 0.12	0.15 ± 0.15	0.7 ± 0.3	0.47 ± 0.13	4
7.5/7.0	0.2 ± 0.1	0.23 ± 0.16	0.9 ± 0.5	1.5 ± 0.5	4

## Discussion

Several ion channels are gated or allosterically modulated by pH^40^. For some channels (e.g. two P-domain K^+^ channels), the amino-acid residues that convey pH sensitivity, presumably by titration of acidic or basic side chains, are well characterized^41,42^. However, Hv1 is modulated by ΔpH rather than pH_i_ or pH_o_ itself. Although some mutations have been reported to affect ΔpH sensing of Hv1^26,28,30^, a clear identification of amino-acid residues that constitute the ΔpH sensor(s) is lacking. Here, we provide direct evidence that the S4 segment alters its conformation in response to changes in ΔpH, suggesting that S4 serves as both voltage- and ΔpH-sensor.

During voltage-dependent gating, Hv1 proceeds through multiple transitions^36,37,43–45^. VCF recordings show that membrane depolarization moves the S4 voltage sensor to an activated state, followed by a second transition that leads to the open state^36^. Taking advantage of precise intra- and extracellular pH control in the PCF configuration, we show that the transitions monitored at S4 are, except for the activation kinetics, relatively similar for different symmetric pH conditions (Fig. 2G, ΔpH = 0), suggesting that the voltage dependence of voltage-sensor motion, like voltage-dependent gating, does not depend on pH itself. By contrast, asymmetric pH conditions can lead to a drastic change of the F_ignal_ on S4 (Figs. 3 and 5), suggesting that voltage and ΔpH are the key stimuli to drive S4 conformational changes.

Recently, two studies investigated Hv1 mutants with diminished ionic currents and could identify gating currents, which reflect the movement of charged amino-acids side chains across the electric field of the membrane^44,45^. Interestingly, gating currents of human Hv1-W207A-N214R were shown to be sensitive to pH_o_: extracellular acidification by one pH unit shifts the gating charge–voltage relationship towards positive membrane potentials by 40 mV^45^. Because S4 contains the majority of gating charges^43^, the pH_o_ sensitivity of gating currents suggests that the S4 conformation depends on pH_o_. Our results show that the S4 conformation is also sensitive to pH_i_. This is in agreement with a previous study that suggested that the gating transitions for Hv1 activation depend on pH_i_^37^.

**Figure 7:**
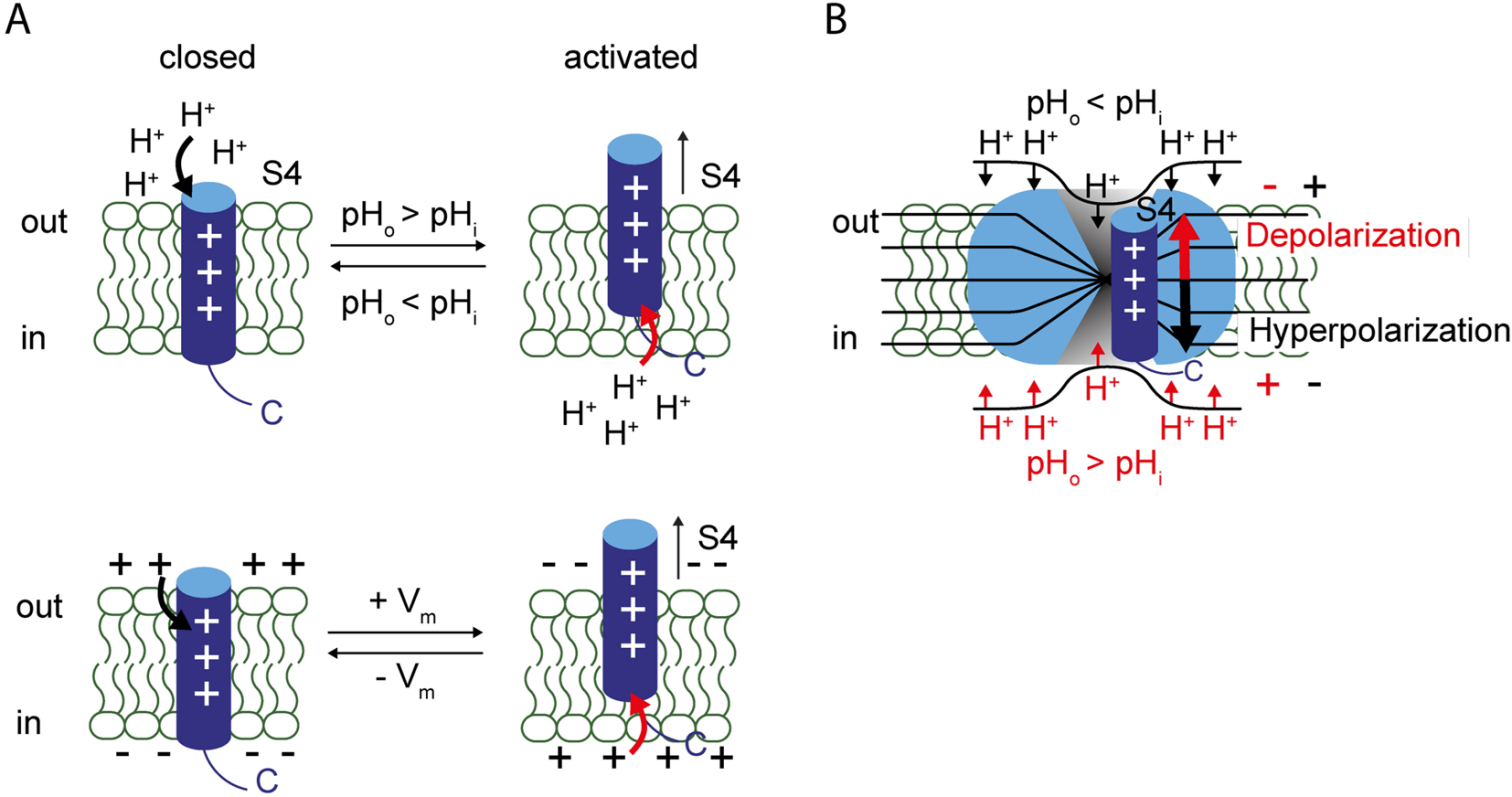
Both ∆pH and the membrane potential control S4 conformation. (**A**) proposed S4 conformation in the membrane as a function of either ∆pH (top) or voltage (bottom). Protons on one side of the membrane move S4 to the opposite side of the membrane (top), similar to the effect of membrane voltage (bottom). S1-S3 are omitted for clarity. “ + ” signs denote the charged arginines in S4. (**B**) cartoon depicting how S4 position is determined by both voltage and ∆pH across the membrane. Protons might exert electrostatic forces on Hv1, i.e. by protonation of a water wire in the VSD. The position of the mobile S4 segment depends on both, the electrochemical potential for protons and the membrane potential: excessive protons at the extracellular side (pH_o_ < pH_i_) and/or hyperpolarization push S4 to the intracellular side, stabilizing the closed state. Excessive protons at the intracellular side (pH_o_ > pH_i_) and/or depolarization push S4 to the extracellular side, stabilizing the activated state.

Taken together, these results are consistent with the idea that excessive protons at the intracellular side (low pH_i_) or a positive membrane potential push S4 to the extracellular side; excessive protons at the extracellular side (low pH_o_) or a negative membrane potential push S4 to the intracellular side (Fig. 7A). Therefore, we suggest that both ΔpH and voltage determine the position of S4 in the membrane (Fig. 7B). Hv1 might enter states by changes in ΔpH that are different from states by changes in membrane potential. Whether ΔpH sensing is restricted to only certain conformational states, remains unclear and thus a subject for future studies. Our PCF experiments provide strong evidence that S4 conformation can be changed by pH_i_ in the resting-state (Figs. 3, 5). Our proton uncaging experiments show that the open state is sensitive to changes in pH_o_ (Fig. 4A). In a previous study of human Hv1, mutation of the first of the three arginines of the S4 segment to histidine („R1H”) creates an additional, hyperpolarization-activated proton conductance, termed shuttle conductance^46^. The shuttle conductance was found to be pH_o_-sensitive, which might reflect the ΔpH-sensitivity of the S4 segment for transitions between resting (non-open) states at negative membrane potentials.

Different molecular mechanism of ΔpH sensing are currently under debate^47^. Protonation or deprotonation of amino-acid residues can induce conformational changes in proteins. In Hv1, there might be distinct, protonable amino-acid residues serving as pH sensors at either side of the membrane for detecting pH_o_ and pH_i_, respectively^24^. S4 contains only three arginines as potentially titratable amino-acid side chains, and those might be difficult to deprotonate under physiological conditions. If any of those arginines were deprotonated over the tested pH range, we would expect a significant change in voltage sensitivity, which should result in shallower slopes of the GVs. However, slopes for different pH conditions did not change significantly (Table 1). Alternatively, the negatively charged amino-acid residues in transmembrane segments S1–S3, which serve as countercharges of the arginine residues, might participate in pH sensing^48^; titration of those residues might weaken their interaction with the arginine residues and, therefore, influence the S4 conformation. However, because ΔpH and not the pH itself determines gating, sensors for pH_o_ and pH_i_ need to be influenced by or functionally linked to each other. The strict dependence of V_1/2_ on ΔpH, which is remarkable linear over a large pH range^26^, argues against titratable amino-acid residues as pH sensors: the pK_a_s of the intra- and extracellular sensor would need to be coupled in a symmetric fashion which appears implausible.

It has been suggested that water molecules could be an important component in mediating ∆pH sensitivity. Molecular dynamics simulations suggest that the core of the VSD of Hv1 contains more water molecules than the VSD of classical voltage-gated ion channels^26^. Indeed, several solvent accessible amino-acid residues are located deep inside the crevice of the VSD^49^. The water molecules might form a robust water wire network with limited mobility within the VSD. Protonation of this network could lead to a rearrangement of the hydrogen bonding and exert electrostatic forces that change S4 conformation^26^. Such a mechanism is not localizable to the Hv1 channel itself; it would be a distributed effect acting on the channel. A previous study on Zn^2+^ sensitivity in Hv1 provides evidence for allosteric coupling between intra- and extracellular residues induced through coulombic interactions^50^. This supports the idea that changes in electrostatic forces might be processed over a long distance across the membrane.

We did not find evidence for direct ∆pH-induced conformational changes of the S1 segment, reinforcing the idea that the mechanisms of ΔpH- and voltage-sensing are intimately linked to each other via the S4 voltage sensor. However, we cannot rule out the possibility that the S4 segment is coupled to other -still unknown- ∆pH-sensing elements. Further studies are therefore needed to further characterize the ∆pH-induced conformational changes of the S4 segment as well as possible additional conformational changes involving other segments.

It is not known whether only the S4 segment of Hv1 is ΔpH sensitive, or whether the S4 segments of other voltage-gated ion channels are also ∆pH sensitive. The ΔpH-induced S4 movement during non-conducting states suggests that actual proton permeation is not a prerequisite for ∆pH sensing. Possibly, changes in ∆pH can induce motions in the VSDs of other voltage-gated ion channels that do not necessarily couple to channel opening and therefore might have escaped from being noticed in electrophysiological recordings. The higher water densities in Hv1 compared to other VSDs might play a key role for ∆pH sensing by enabling an interaction of protons with S4.

Our data suggest that the functional coupling between pH_o_ and pH_i_ sensor(s) is provided by the mobile S4 voltage sensor, serving as a whole as the “∆pH-sensing element”. In this setting, the electrochemical potential for protons across the membrane, together with the membrane potential, determines the position of S4 in the membrane (Fig. 7B), and thereby sets the voltage of half-aximal channel activation. Of note, the ∆pH-induced shift of V_1/2_, reported to amount to − 0 mV/∆pH unit in the literature and to − 47 to − 57 mV/∆pH unit for the channels investigated in our study, is close to the electrochemical potential for protons (–59 mV/∆pH unit), suggesting an immediate link between gating and the permeant ion. Deviations might arise from differences between ion concentration and activity. Mutations that limit water exposure to S4 should decrease ∆pH sensing; those mutations, however, are likely to limit conduction as well. Clearly, the identification of the detailed molecular mechanism behind ∆pH sensing of Hv1 requires further studies using electrophysiological, proton uncaging, and fluorometric techniques on mutants of Hv1 as well as modeling approaches that take the potential profiles around Hv1 into consideration.

## Data Availability

The datasets generated during and/or analyzed during the current study are available from the corresponding author on reasonable request.
